# Ectopic A-lattice seams destabilize microtubules

**DOI:** 10.1038/ncomms4094

**Published:** 2014-01-27

**Authors:** Miho Katsuki, Douglas R. Drummond, Robert A. Cross

**Affiliations:** 1 Division of Biomedical Cell Biology, Warwick Medical School, University of Warwick, Coventry CV4 7AL, UK; 2 Present address: RIKEN Center for Life Science Technologies, Division of Genomic Technologies, Yokohama, Kanagawa 230-0045, Japan

## Abstract

Natural microtubules typically include one A-lattice seam within an otherwise helically symmetric B-lattice tube. It is currently unclear how A-lattice seams influence microtubule dynamic instability. Here we find that including extra A-lattice seams in GMPCPP microtubules, structural analogues of the GTP caps of dynamic microtubules, destabilizes them, enhancing their median shrinkage rate by >20-fold. Dynamic microtubules nucleated by seeds containing extra A-lattice seams have growth rates similar to microtubules nucleated by B-lattice seeds, yet have increased catastrophe frequencies at both ends. Furthermore, binding B-lattice GDP microtubules to a rigor kinesin surface stabilizes them against shrinkage, whereas microtubules with extra A-lattice seams are stabilized only slightly. Our data suggest that introducing extra A-lattice seams into dynamic microtubules destabilizes them by destabilizing their GTP caps. On this basis, we propose that the single A-lattice seam of natural B-lattice MTs may act as a trigger point, and potentially a regulation point, for catastrophe.

## Abstract (web summary)

Microtubules are tubes in which helical symmetry is broken at a single ‘A-lattice’ seam. Katsuki *et al.* show that microtubules containing additional A-lattice seams exhibit decreased stability, and propose that such seams may act as trigger points for microtubule catastrophe.

## Graphical abstract

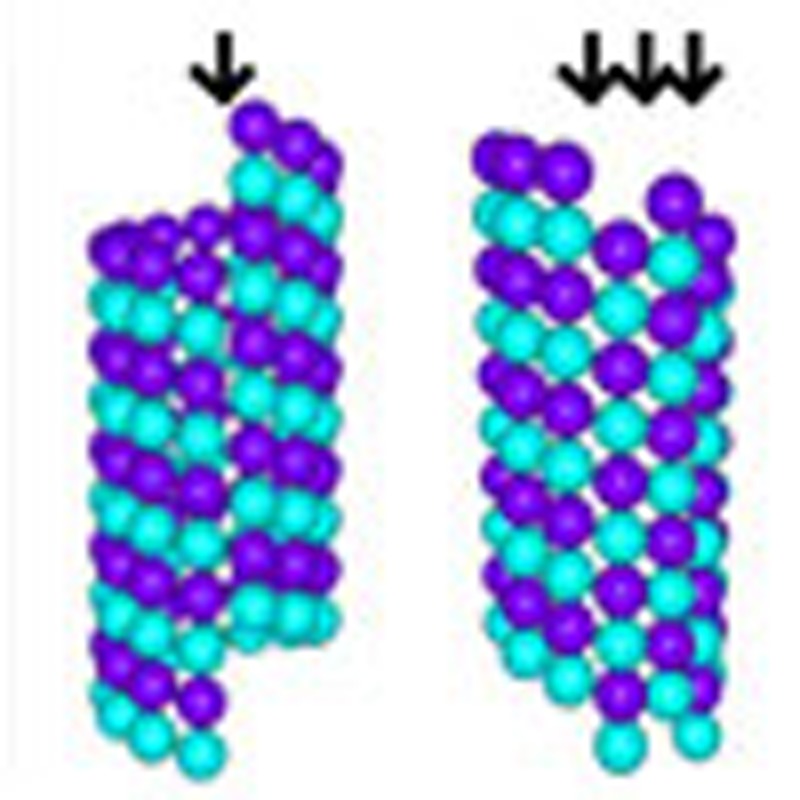

Microtubules (MTs) play a central role in the self-organization of eukaryotic cells, driving directional transport of cellular components either by using their own dynamics, or by serving as rails for cargo-carrying motor proteins. MTs self-assemble from α–β tubulin heterodimers to form hollow tubes of ∼25 nm diameter^1^. MTs assembled from GTP–tubulin undergo cycles of spontaneous growth, catastrophe, shrinkage and rescue. This behaviour is termed dynamic instability^2^ and is driven by GTP hydrolysis^3^. GTP–tubulin subunits add to the growing MT tip and form a stabilizing cap^4^. GTP–tubulin in the cap converts continually to GDP–tubulin via hydrolysis and phosphate release. The GDP core of the MT is unstable compared with the GTP cap. Loss of the cap in a catastrophe event exposes the unstable GDP core, which then rapidly shrinks unless growth is re-established in a rescue event. While this behaviour is well established, the detailed molecular mechanism of catastrophe, by which MTs lose their stabilizing cap and convert from steady growth to sustained shrinkage, is much less clear.

Catastrophe, the process of conversion from sustained growth to rapid shrinkage, ultimately results in the breaking of lateral bonds between protofilaments (PFs), leading to rapid shrinkage of the MT with coupled unpeeling of outwardly curved GDP–tubulin PFs^5^,^6^. Catastrophe was originally reported to be a completely random process in MTs assembled from pure tubulin^7^, but recent evidence suggests that catastrophe frequency increases with the age of the MT, consistent with a multi-step process^8^,^9^. The extent and detailed structure of the GTP cap are controversial^10^. Classical rapid dilution experiments indicate that a relatively shallow cap of GTP tubulin is sufficient to stabilize MTs^11^,^12^,^13^,^14^. However, there is evidence that GTP tubulin islands can, at least sometimes, persist into the core of the MT^15^. Recent models propose that the portion of the GTP tubulin cap that provides structural stability may be shorter than the region containing GTP tubulin (reviewed in ref. 16). The nature of the tubulin interactions within the MT lattice that stabilize the GTP cap also remains unclear. It is possible that GTP tubulin molecules themselves form more stable lateral contacts^17^ but they may also promote the formation of lateral contacts indirectly, by forming more stable longitudinal contacts^18^. Recent molecular dynamic simulations suggest a combination of strengthened longitudinal and lateral bonds^19^.

MTs in mammalian cells typically contain 13 straight PFs arranged in the B-lattice^20^ with a single-seam of A-lattice contacts^21^,^22^,^23^ (Fig. 1). By altering PF number, perfectly helically symmetric B-lattice MTs with no A-lattice seam can be built^20^ and do occur, both *in vitro*^24^ and *in vivo*^25^,^26^,^27^,^28^, but these are the exception rather than the rule. Pure A-lattice MTs with straight PFs are also possible^20^ and have been observed *in vitro*^29^ but not *in vivo*. Instead, there appears to be a biological drive to build B-lattice MTs that break symmetry by including an A-lattice seam. Why is this? One possibility is that A-lattice seams have a stability different from that of the B-lattice^30^. If MTs assemble via the rolling of a nascent B-lattice sheet of PFs into a tube^6^, then the A-lattice seam is the last inter-PF interface to close, suggesting that it might be the weakest part of the MT. There is modelling evidence suggesting that A-lattice PF contacts are less stable than B-lattice PF contacts^31^. MTs have also been observed to split along a specific PF contact^20^ and cold-induced disassembly can cause MTs to open into flat sheets, suggesting that MTs fail along a single, cold-labile seam^32^. However, since tube closure brings enhanced stability compared with an open sheet, the A-lattice seam, once formed, might be no less stable, or even more stable, than the rest of the tube. The ends of MTs assembling *in vivo* have a flared structure, suggesting no preferential formation of any particular lateral contacts and therefore little difference in the stability of seam and sheet contacts^33^. Su and Downing^34^ showed by cryoelectron microscopy that the M, H1′–S2 and H2–H3 tubulin loops form bridging density between neighbouring PFs in MTs that is very similar in the A-lattice seam and the main B-lattice. This suggests that the mechanical properties of the A-lattice seam may be similar to those of the rest of the lattice, with similar salt bridges forming in α–α, β–β and α–β lateral contacts.

A direct experimental test of the influence of A-lattice seams on MT catastrophe has hitherto been lacking, because it has not previously been possible to vary the A-lattice content of MTs. Spontaneous *in vitro* assembly of MTs with either no A-lattice seams^24^ or multiple A-lattice seams^30^ occurs too rarely to be experimentally useful. MT assembly in high salt can form MTs with up to 50% A-lattice contacts, but these MTs also have only 10 PFs^35^ and it is unclear how this would affect their properties. We recently found that the *S. pombe* EB protein Mal3, when added in high concentration during nucleation can drive the assembly of A-lattice-enriched 13 PF MTs, with a population mean of ∼50% A-lattice content^29^. Here we have used Mal3 as an experimental tool to create A-lattice-rich MTs and measure their dynamic properties. Mal3 was removed before measurements were made and we do not address here the mechanism by which Mal3 produces A-lattice rich MTs. We find that MTs with extra A-lattice content show more frequent catastrophes than normal B-lattice MTs, consistent with an earlier proposal that A-lattice seams provide a regulation point for MT instability^36^.

## Results

### Kinesin surface clamp-release assay

**Figure 1:**
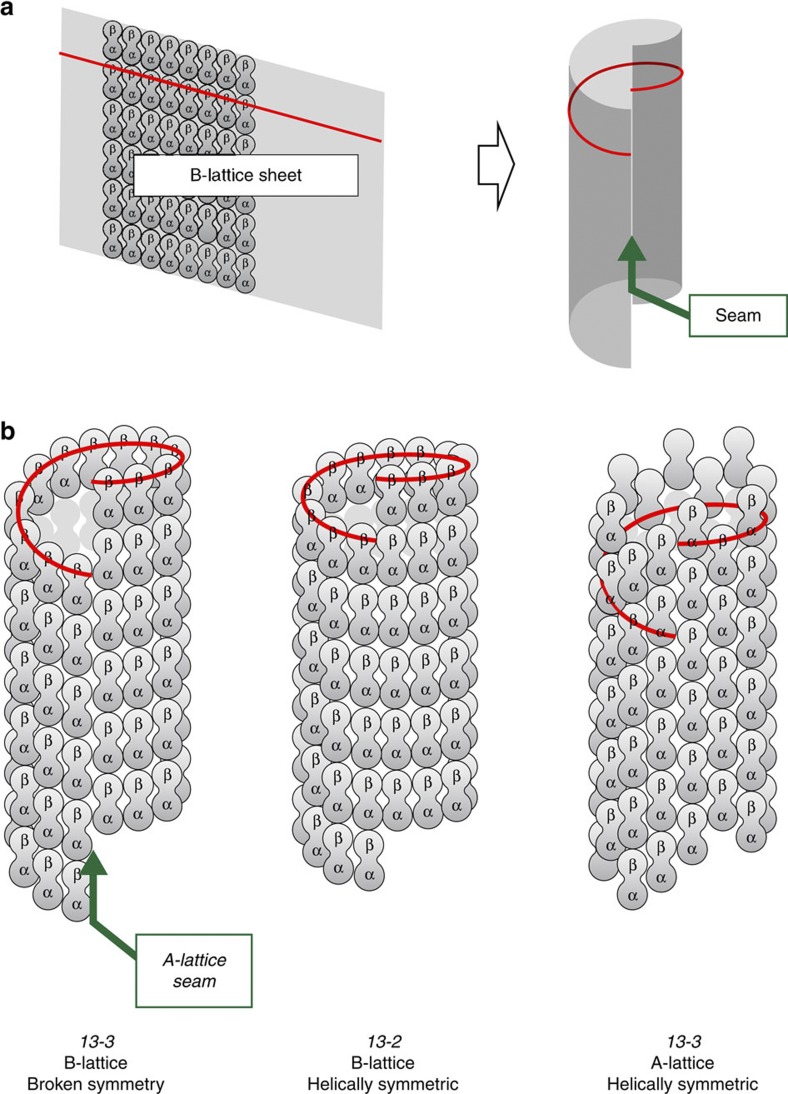
MT lattice packing of tubulin heterodimers. Adjacent PFs within a MT B-lattice have a relative axial shift of 0.9 nm forming major α–α and β-β lateral contacts between tubulin heterodimers (**a**). The flat sheet of PFs can close to form a tube. The MT lattice geometry is determined by the number of PFs and the start number of helices that can be drawn through the tubulin subunits around the tube (red line). In a three-start helix three independent spirals of tubulin subunits are required to fill the MT lattice. For a B-lattice 13 PF three-start MT (13–3) tube closure is only possible if a seam forms containing A-lattice contacts with a 4.9-nm axial shift between adjacent PFs and α–β lateral connections (**b**). For 13 PF MTs helically symmetric tubes without any seams can form from 13–2 B-lattice or 13–3 A-lattice MTs. The 13–3 B-lattice with a single A-lattice seam is the only B-lattice MT where the PFs are parallel with the longitudinal MT axis. In the other types of B-lattice a tilt of the PFs axes relative to the MT axis is required to achieve registration of the tubulin subunits.

**Figure 2:**
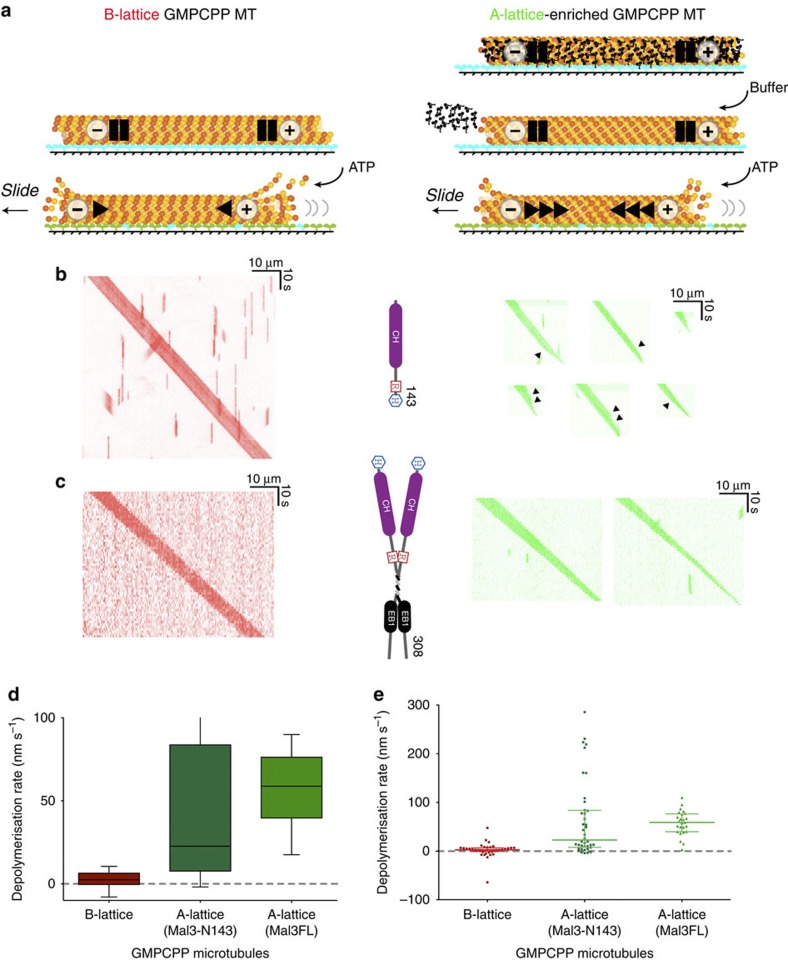
Stability of A-lattice seam-enriched GMPCPP MTs. MT seeds were assembled from Alexa fluorophore-labelled pig brain tubulin using the slowly hydrolysable GTP analogue GMPCPP to create B-lattice MTs with a single A-lattice seam. Co-assembly with Mal3 monomer Mal3–N143 or dimer Mal3FL formed A-lattice-enriched MTs. After pelleting through a glycerol cushion MTs were attached to a flow cell surface coated with rat kinesin-1 motor protein rKin430 and the flow cell flushed with buffer to remove any residual Mal3. ATP was then added to activate the kinesin-1 MT translocase activity and release the kinesin clamp on the MTs. Blue: rigor bound kinesin heads. Green: detached kinesin heads. (**a**). MT images were recorded by fluorescence microscopy and kymographs created from the movies of translocating MTs. B-lattice single-seam MTs (red kymographs) were compared with A-lattice-enriched MTs (green kymographs) assembled with either Mal3 monomer Mal3–N143 (**b**) or Mal3FL dimer (**c**). B and A-lattice-enriched MTs in (**b**) were both labelled with Alexa-488 fluorophore and recorded separately. The A- and B-lattice MTs in (**c**) were recorded in the same flow cell. To do this, A-lattice-enriched MTs were labelled with Alexa-488 and B-lattice single-seam MTs dual labelled with Alexa-680 and Alexa-488. A single shrinkage rate was calculated for each MT from the total decrease in MT length and the distributions were plotted as box and whisker (box: median and interquartile range, whisker: 10–90% (**d**) or as a scatter plot of all data points with median and interquartile ranges (**e**). The median shrinkage rates are significantly different (Kruskal–Wallis test (*H*=49.139, df=2, *P*=2.14 × 10^−11^) with median A-lattice rates of 22.6 nm s^−1^ (*n*=40) for Mal3-N143 and 58.8 nm s^−1^ for Mal3FL (*n*=25), significantly faster (Dunn’s post-test *P*<0.001) than the B-lattice MT shrinkage at 2.5 nm s^−1^ (*n*=43). Variable rates of shrinkage were observed within the same MT assembled with Mal3–N143. Changes in rate are indicated in **b** (black arrowheads). Phases with distinct shrinkage rates extended along the MTs for lengths of 0.22, 2.67 μm; 0.69, 1.56 and 1.94 μm in the top panels; 1.08, 1.23, 1.50 μm; 0.27, 1.26, 1.18 μm and 0.76, 1.33 μm in the lower panels.

To examine the properties of MTs with extra A-lattice content we developed a kinesin motor surface clamp-release assay. We found that MT shrinkage can be inhibited by clamping a MT to a kinesin-coated coverslip (Fig. 2a). We used this kinesin-clamp assay to explore the effects of increased A-lattice content on MT stability. MTs assembled from pure tubulin contain B-lattice with a single A-lattice seam, which we will refer to as B-lattice single-seam MTs. Including Mal3 during MT assembly creates a population of MTs with varying degrees of A-lattice seam enrichment including some containing pure A-lattice (Fig. 1b)^29^, which we will refer to as A-lattice-enriched MTs. Following assembly, MTs are bound to a kinesin-coated surface in the absence of ATP. Binding the MTs to a rigor kinesin surface stabilizes them and allows us to exchange buffers, wash away residual Mal3 and alter the free tubulin concentration. Removing Mal3 is necessary because lattice-bound Mal3 can stabilize MTs against disassembly^37^. The advantage of using kinesin molecules as clamps to stabilize the MTs is that we can loosen the kinesin clamp by adding ATP.

### A-lattice enrichment causes GMPCPP MTs to shrink faster

GMPCPP is a slowly hydrolysable GTP analogue^3^, therefore GMPCPP MTs are models for the stabilizing GTP cap of dynamic MTs. Pig brain tubulin was assembled either alone to generate B-lattice GMPCPP MTs containing a single A-lattice seam or with a short monomeric Mal3 construct Mal3–N143 (Mal3 CH-domain) to generate A-lattice-enriched GMPCPP MTs with an average of ∼50% A-lattice content^29^. Mal3 formed A-lattice-enriched MTs with both *S. pombe* and pig brain tubulin^29^. We selected pig brain tubulin for these assays as it is better stabilized by GMPCPP, making analysis of A-lattice effects easier. Excess Mal3 was removed by centrifugation through a glycerol cushion and the MTs were examined using the kinesin surface-clamp assay, binding the MTs to a rigor kinesin surface and flushing with ATP-free buffer to remove any residual Mal3 (Fig. 2a). When bound to the kinesin surface in rigor conditions, both the A-lattice-enriched and B-lattice single-seam GMPCPP MTs were stable. On flushing with ATP-containing buffer the kinesin started to walk and both types of GMPCPP MTs translocated over the kinesin-coated surface (Supplementary Movie 1). The translocating GMPCPP B-lattice single-seam MTs remained relatively stable (Fig. 2b–e) with an overall median shrinkage rate of 2.5 nm s^−1^ (calculated as the sum of shrinkage at both MT ends). B-lattice single-seam seeds containing Alexa-480 fluorescent label (Fig. 2b) had similar stability to dual Alexa-680 and Alexa-480 fluorescently labelled seeds (Fig. 2c). By contrast, A-lattice-enriched Alexa-480-labelled GMPCPP MTs assembled using Mal3-N143 (Fig. 2b,d,e) shrank faster, with median shrinkage rates of 22.6 nm s^−1^. These A-lattice-enriched seeds were created using the same single-headed Mal3 construct and in similar conditions to those of the original study^29^. We also investigated whether a double-headed full-length Mal3, which has a higher MT-binding affinity^29^, used at a higher concentration of 50 μM would also affect MT seed stability. The Mal3FL seeds produced had an even faster median shrinkage rate of 58.8 nm s^−1^ (Fig. 2c–e). There was more variation in the observed shrinkage rates with Mal3-N143 than with Mal3FL (Fig. 2e) and some spontaneous breakage of Mal3-N143 assembled MTs was also observed. MTs formed in the presence of dimeric Mal3FL were preferred for subsequent experiments since although the peak shrinkage rates observed were lower (Fig. 2e), the median shrinkage rates were higher. To control for any effect from residual Mal3 in the flow cell, experiments employing Mal3FL seeds used a mixture of dual-labelled B-lattice single-seam GMPCPP MTs and single-labelled A-lattice-enriched GMPCPP MTs in the same flow cell (Fig. 2c, Supplementary Movie 2). Again only the A-lattice-enriched MTs shrank rapidly. For A-lattice-enriched (Mal3–N143) GMPCPP MTs but not for B-lattice GMPCPP MTs, we saw episodic shrinkage, occurring at a constant rate over a distance up to ∼2.5 μm along the MT (Fig. 2b), followed by an abrupt switch to a different rate, with this new rate again typically sustained for several micrometres. This suggests that A-lattice-enriched MTs contain distinct regions that extend for several micrometres along the axis. The data show that the median shrinkage rate of GMPCPP A-lattice-enriched pig brain MTs is increased >20-fold compared with control B-lattice single-seam GMPCPP MTs (Fig. 2d,e).

### Kinesin translocates A-lattice-enriched MTs at normal rates

**Figure 3:**
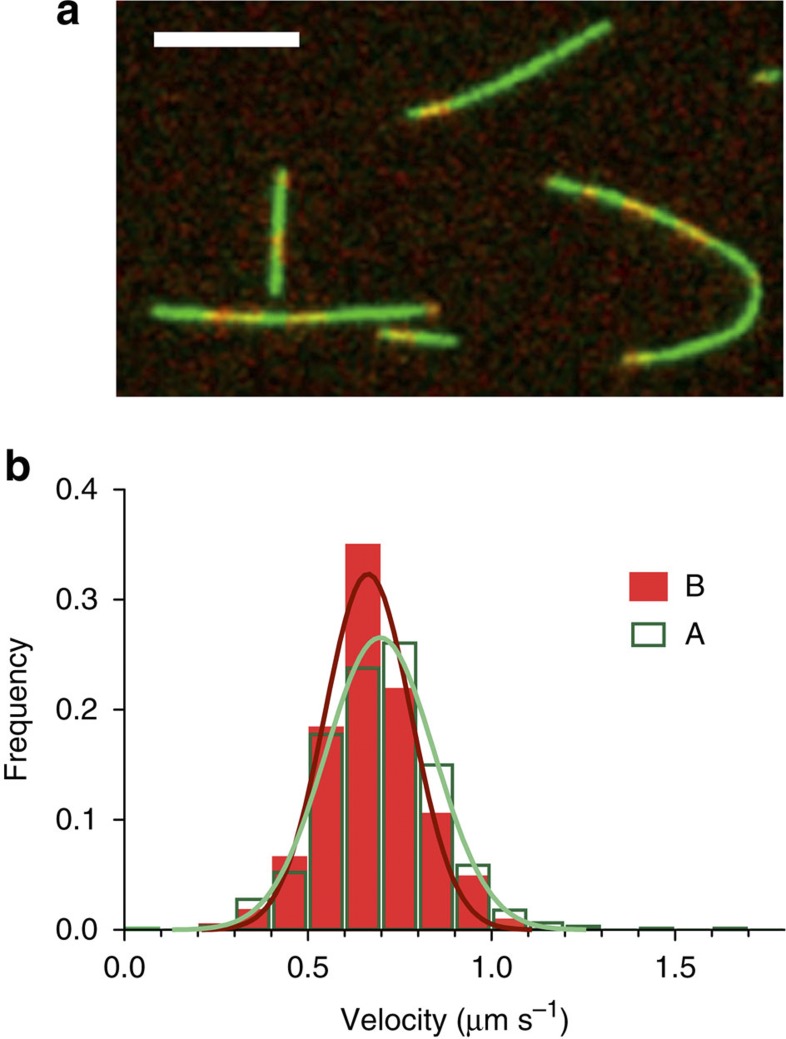
Motility and shrinkage of A-lattice seam-enriched MTs. Segmentally labelled (Lemur-tailed) A-lattice-enriched MTs with alternating Alexa-680 (red) and Alexa-488 (green) labelling were created by co-assembly of Alexa-labelled pig brain tubulin and 50 μM Mal3FL with GMPCPP. MTs were imaged moving over a surface of kinesin-1 motor protein using fluorescence microscopy (**a**); scale bar, 5 μm. The internal MT stripes were used to determine the actual MT velocity and the velocity distribution of B- and A-lattice-enriched MTs were plotted (**b**). The mean velocities of B-lattice MTs were slightly slower at 675±9 nm s^−1^ (229) (mean±s.e.m. (*n*)) compared with A-lattice-enriched MTs at 707±6 nm s^−1^ (614) (Student *t*-test with Welch’s correction for unequal variances, *P*=0.0043). Gaussian fits to the distributions are shown in (**b**) with mean velocities of 660±12 nm s^−1^ (229) in A-lattice and 700±15 nm s^−1^ (614) in B-lattice (mean±s.d. (*n*)).

To control for the possibility that the kinesin surface interacts differently with B-lattice single seam and A-lattice-enriched multi-seam MTs, we checked for a difference in the kinesin-driven sliding velocity for the two types of MT. However, since in our assay the A-lattice-enriched GMPCPP MTs shrank rapidly while sliding over the kinesin surface, we could not immediately compare their velocity with that of the normal B-lattice single-seam MTs. We therefore used ‘lemur tail’ segmentally marked A-lattice-enriched GMPCPP MTs assembled using Mal3FL. Lemur tails are formed by the spontaneous end-to-end annealing of fluorescently labelled Alexa-488 pig brain tubulin MTs nucleated by Alexa-488 and Alexa-680 dual-labelled stabilized pig brain MT seeds (Fig. 3a). The mean sliding velocity of these lemur tail A-lattice-enriched GMPCPP MTs was similar to that of B-lattice single-seam GMPCPP MTs (707±6 nm s^−1^ (614) versus 675±9 nm s^−1^ (229) mean±s.e.m. (*n*); *P*=0.0043, *t*-test with Welch’s correction for unequal variances) (Fig. 3b), indicating that kinesins interact similarly with the two lattices.

### A-lattice-enriched seeds increase MT catastrophe frequency

**Figure 4:**
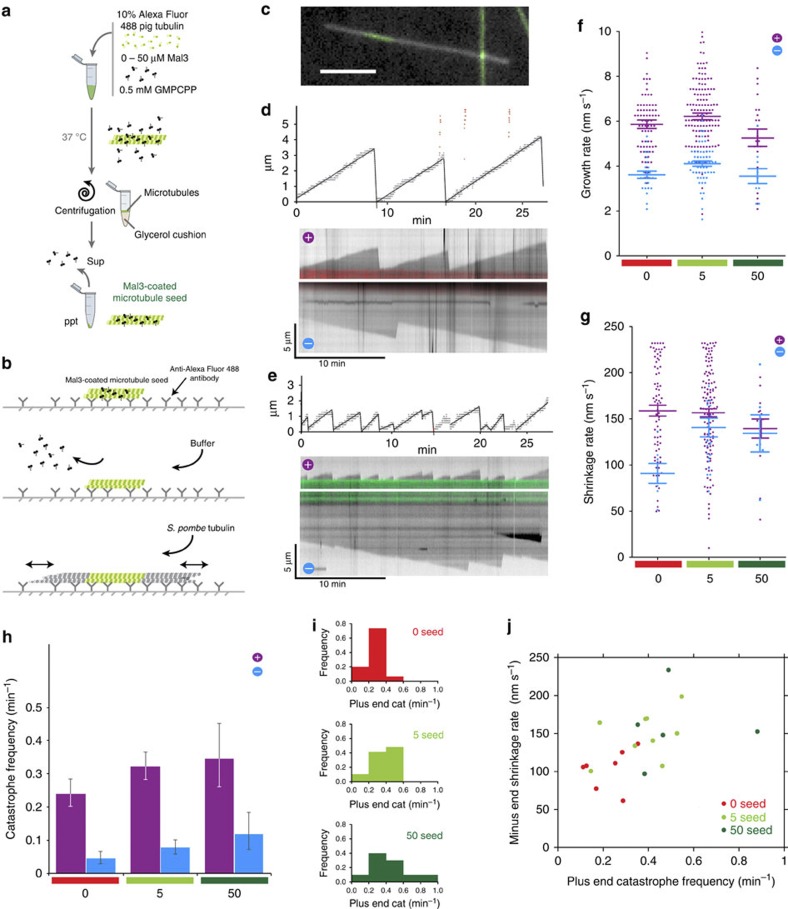
Effect of ectopic A-lattice seams on MT dynamics. B-lattice single-seam MT seeds were assembled using pig tubulin labelled with Alexa-488 fluorescent dye and the GTP analogue GMPCPP. A-lattice-enriched seeds were co-assembled from Alexa-488 pig tubulin, GMPCPP and 5 μM or 50 μM Mal3FL and pelleted through a glycerol cushion to remove excess Mal3 (**a**). Seeds were attached to a flow cell surface using Anti-Alexa-488 antibody, the flow cell flushed with buffer to remove any residual Mal3 and then free *S. pombe* tubulin and GTP were added to enable growth of dynamic GTP MTs (**b**). The location of the Alexa-488-labelled seeds (green) was visualized using epifluorescence microscopy and the dynamic unlabelled MT ends by dark-field microscopy (grey) (**c**). Kymographs of MTs were recorded from B-lattice (**d**, red) and A-lattice-enriched MTs (**e**, green), the end location of the MTs determined automatically, and the MT dynamic parameters of growth (**f**) and shrinkage (**g**) rates together with catastrophe frequency (**h**) determined and plotted for the fast plus end (purple) and slow minus end (blue) of MTs nucleated by GMPCPP tubulin seeds (red) or seeds co-assembled with 5 μM (light green) or 50 μM (dark green) Mal3FL. Error bars in (**f**) and (**g**) show s.e.m. and in (**h**) the Poisson confidence limits (*P*=0.05). Mean rates and frequencies are shown in Table 1. (**e**) is an example of plus end enhanced catastrophe frequency often observed in A-lattice-enriched MTs that was infrequently seen in B-lattice single-seam MTs (**d**). The distributions of catastrophe frequencies for MTs growing from individual seeds were also plotted (**i**). The minus end shrinkage rate for individual seeds was then plotted against the plus end catastrophe frequency for the same seed using the pooled data from the 0, 5 and 50 μM seeds (**j**). The minus end shrinkage and plus end catastrophe of the pooled data have a significant correlation (Spearman coefficient *r*=0.6039, *P*=0.0037). Scale bar in (**c**) is 4 μm.

Dynamic MTs were polymerized from GMPCPP-stabilized MT seeds to determine whether the MT dynamics were altered by A-lattice enrichment of the seeds. We had previously studied the effect of Mal3 on the dynamics of *S. pombe* MTs nucleated by B-lattice single-seam pig tubulin seeds^37^, therefore we utilized this well-characterized system. Seeds containing extra A-lattice were assembled by including 5 or 50 μM of Mal3FL at the nucleation step^29^. Excess Mal3 was then removed before dynamics were measured by pelleting through a glycerol cushion (Fig. 4a). Alexa-488-labelled seeds were attached to a flow cell surface using anti-alexa-488 antibodies, which stabilize the GMPCPP seeds similarly to rigor binding of kinesin. Flow cells were flushed with buffer to remove any residual Mal3 or glycerol and removal of residual Mal3 was confirmed in control experiments using GFP–Mal3 fusion protein, which was undetectable after the flushing steps. Purified unlabelled single-isoform *S. pombe* tubulin and GTP were then flowed in. The added tubulin polymerizes from the seeds to form dynamic MTs, which are only anchored to the surface through the antibody-bound seed (Fig. 4b). Seed position was recorded using epifluorescence microscopy, and the entire dynamic MT was imaged by dark-field microscopy using an illumination wavelength to which the fluorophore in the seeds is insensitive (Fig. 4b,c, Supplementary Movie 3). Kymographs were created and the MT ends automatically tracked (Fig. 4d,e). The data show that the plus-end catastrophe frequency of MTs growing from A-lattice rich MT seeds is enhanced at least 1.5-fold over those growing from B-lattice single-seam seeds with a single A-lattice seam. The minus-end catastrophe frequency is also enhanced, by threefold (Fig 4h, Table 1). We examined the catastrophe frequency of dynamic MTs grown from individual seeds. The catastrophe frequencies of MTs grown from B-lattice single-seam seeds were more tightly clustered than with the 5 or 50 μM Mal3FL seeds, for which higher catastrophe frequencies were more common (Fig. 4i). Plus-end shrinkage rates following catastrophe were not significantly different. The absence of an effect on shrinkage is not a detection artefact, since shrinkage of the MTs in Fig. 4e would still be easily detectable over several data points at more than twice the actual rates measured. Minus end shrinkage is 1.5 times faster in A-lattice-rich MTs than in B-lattice MTs (Fig. 4g, Table 1). MTs with faster minus-end shrinkage rates were more likely to grow from seeds with higher plus-end catastrophe frequencies (Fig. 4j).

We also tested whether very low residual amounts of Mal3 in the dynamics assay might account for the increase in catastrophe frequency. Deliberate addition of low concentrations of Mal3FL to dynamic MTs nucleated by B-lattice single-seam seeds had no effect on plus-end catastrophe frequency at any of the concentrations tested and tended to slow shrinkage rates (Table 2), as previously reported^37^.

### A-lattice-enriched seeds do not affect MT growth rates

In the same MT dynamics assays used to measure catastrophe frequency we found that dynamic *S. pombe* MTs grow at similar rates from A-lattice-enriched GMPCPP seeds and B-lattice single-seam GMPCPP seeds (Fig. 2d–f; Table 1). In both cases, plus-end growth was about twice as fast as minus-end growth (Fig. 2f; Table 1).

### MT dithering

**Figure 5:**
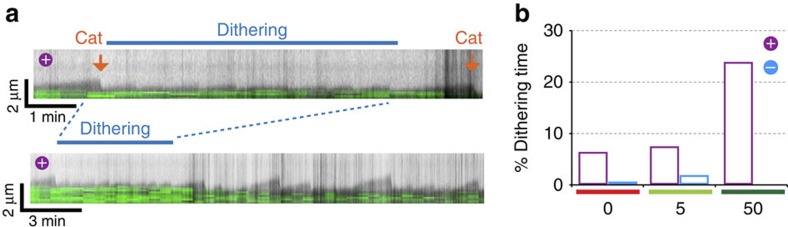
MT dithering in A-lattice seam-enriched MTs. (**a**) A kymograph of the plus-end of a dynamic *S. pombe* MT grown from an A-lattice-enriched MT seed. Periods of rapid short fluctuations (<3 pixels equivalent to <384 nm) (blue bar, enlarged section of kymograph) appear as abortive elongations from the MT seed (green). (**b**) The % of total observation time spent in periods of such MT dithering at the plus (purple) and minus (cyan) MT ends rather than sustained growth or shrinkage was determined for the dynamic *S. pombe* MTs in Fig. 4 and plotted for dynamic MTs nucleated by B-lattice (red) or A-lattice-enriched seeds co-assembled with either 5 μM (light green) or 50 μM Mal3FL (dark green).

A-lattice enrichment of MT seeds not only promoted catastrophe of the dynamic MTs, it also markedly increased the length of time required for the regrowth of MTs following catastrophe and shrinkage back to the nucleating seed. Often, MTs growing from A-lattice-enriched seeds started to grow, but then immediately underwent catastrophe and shrank (Fig. 5a, Supplementary Movie 4). In these instances the amount of growth corresponded to <3 pixels in our images (∼0.384 μm) and was too small to quantify accurately. Since we cannot characterize this behaviour unambiguously we have classified it as MT dithering. The plus ends of MTs nucleated by A-lattice rich seeds spend four times longer dithering than MTs nucleated by B-lattice single-seam MTs (Fig. 5b, Table 1).

### A-lattice enrichment drives shrinkage of GDP MTs

**Figure 6:**
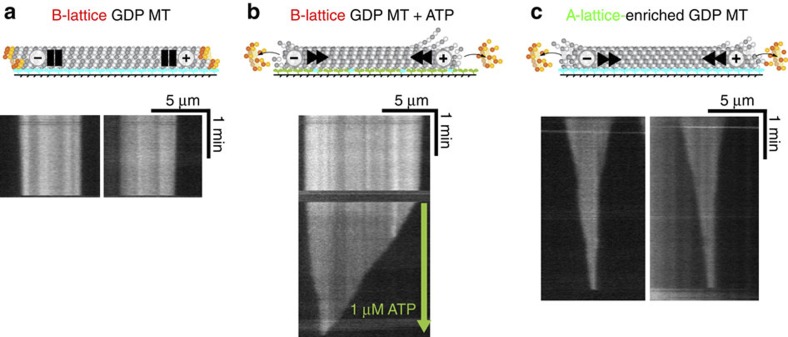
Effect of A-lattice seam enrichment on GDP MTs. Pure *S. pombe* single isoform tubulin MTs were assembled with GTP for B-lattice single-seam MTs (**a**,**b**) or with GTP and Mal3FL for A-lattice-enriched MTs (**c**). MTs were attached to a flow cell surface coated with rKin430, a double-headed construct of rat kinesin-1. The flow cells were flushed with buffer to remove unbound MTs and Mal3, then the MTs were imaged by dark-field microscopy, kymographs created and the shrinkage rates measured (Table 3). The B-lattice single-seam GDP MT was stable when rigor bound to rKin430 kinesin (**a**). Only when the B-lattice single-seam MT flow cell was flushed with buffer containing ATP to enable MT translocation was significant shrinkage observed (**b**). The rigor-bound A-lattice-enriched MTs were unstable and rapidly disassembled before addition of ATP (**c**). Blue: rigor-bound kinesin heads. Green: detached kinesin heads.

We also used our switchable kinesin clamp-release assay to compare directly the stability of A-lattice rich GDP MTs with that of B-lattice single-seam GDP MTs. Dynamic B-lattice single-seam MTs were assembled from pure single-isoform *S. pombe* tubulin using GTP, and then bound to a kinesin-1 coated surface. Free tubulin and GTP were then flushed out with buffer. Removing free tubulin would ordinarily uncap the MTs, exposing their GDP core and triggering rapid end-wise shrinkage. However, since in this assay our uncapped B-lattice GDP MTs are bound via rigor kinesins to a surface, they are essentially stable (Fig. 6a,b). Plus-end shrinkage was a constant 2.68±0.73 (5) nm s^−1^ (mean±s.e.m. (*n*)), ∼60 times slower than the plus-end shrinkage of MTs not tethered by kinesin (Fig. 6a; Tables 1 and 3). Release of the kinesin-1 clamps by addition of 1 μM ATP dramatically increased the shrinkage rate to 55.1±5.1 nm s^−1^ (5) (mean±s.e.m. (*n*)) (Fig. 6b, Table 3). This shrinkage rate was still about three times slower than the plus-end shrinkage rate observed in the MT dynamics assay (Fig. 4, Table 1). However, unlike the dynamics assay where the dynamic MT is not tethered to the surface, the kinesin in the surface-clamp assay with ATP will still transiently attach and detach to the MT, resulting in a weak tethering to the surface and a slowing of the shrinkage rate. A-lattice-rich GDP MTs behaved very differently. On flushing the flow cell with buffer, rapid shrinkage of the MTs was immediately observed (Fig. 6e), even without the addition of ATP. Plus-end shrinkage occurred at 10.68±0.30 nm s^−1^ (5) and minus-end shrinkage at 2.78±0.13 nm s^−1^ (5) (mean±s.e.m. (*n*)). Thus, the GDP core of dynamic A-lattice-enriched MTs is much less stable than dynamic B-lattice single-seam MTs (Table 3). Depolymerization of GDP A-lattice-enriched MTs was too rapid to permit investigation of the effect of adding ATP to the flow cell.

## Discussion

The single-seam of 13-3 B-lattice MTs creates a line of A-lattice PF contacts^20^. The functional effect of this seam on MT stability and dynamics has been unclear. We have exploited our observation that co-assembly of tubulin with Mal3 enriches for the A-lattice^29^ to investigate the functional effect of extra A-lattice seams on MTs. We found that in solution, MTs with extra A-lattice content depolymerized too rapidly to analyse. However, these MTs could be stabilized by attachment to a rigor kinesin surface. The stabilizing effect of this surface could then be reduced substantially by adding ATP to loosen the clamping action of the motor. Using this assay, we found that co-assembly of tubulin with Mal3 dramatically reduced the stability of GMPCPP MTs, so that their median shrinkage rate increased by >20 times, and in some cases by up to 50 times compared with MTs containing a single-seam. Based on our earlier EM work^29^ co-assembly with Mal3 has two structural effects. First, the MTs all have 13 PFs^29^,^38^ as is also found with EB1 co-assembly^39^. However, we do not think that this can explain our observations. The B-lattice MTs would be a population with mixed PF numbers including a high proportion with 13 PFs^24^,^29^ that would be expected to have similarly dramatic shrinkage rates. However, such rapid shrinkage rates were not observed in the B-lattice single-seam seeds assembled from tubulin alone, suggesting that some other factor is altering the behaviour of the Mal3 co-assembled MTs. The second effect of Mal3 co-assembly is to increase the A-lattice content of the MTs from a single-seam to an average of ∼50% mainly as mixed A- and B-lattice MTs, with some MTs containing up to 100% A-lattice^29^. Our results suggest that it is this increase in A-lattice content that is decreasing the MT stability. It is perhaps surprising that such unstable A-lattice-enriched seeds survive the preparation process (Fig. 4a). We speculate that the presence of glycerol and binding of perhaps a few residual Mal3 proteins are sufficient to stabilize some seeds until they are attached to the surface of the experimental chamber. It is possible that we lose a population of seeds with higher A-lattice content. Longitudinal intersubunit contacts are the same in A- and B-lattice, while the lateral inter-PF contacts are different^40^. Our results therefore suggest that the lateral PF contacts are less stable in A-lattice-rich GMPCPP MTs than in B-lattice single-seam GMPCPP MTs.

We found that shrinkage rates varied along the length of A-lattice-enriched GMPCPP MTs co-assembled with Mal3–N143. We speculate that this indicates a variation of A-lattice content along the length of our GMPCPP MTs, with sharp transitions between different zones. Localized zones of additional A-lattice can only be created by a shift in the lateral registration of one PF with its neighbours, creating a defect (a hole) in the MT lattice. Such defects have been observed for B-lattice MTs with variable PF numbers^24^,^41^ and might potentially exist in our MTs. If so, the sharp transitions between zones of constant shrinkage rate suggest that any effects on MT dynamics from such lattice defects are relatively localized.

We find that increasing the A-lattice content of seeds used to nucleate the growth of dynamic MTs enhances catastrophe in the resulting MTs at both their plus and minus ends. Usually, a reduction in MT growth rate correlates with an increase in catastrophe frequency^7^. However, in our case the increase in catastrophe accompanying an increase in A-lattice content in the seed is not accompanied by a change in the MT growth rate. It has been proposed that MT growth rate is strongly influenced by the detachment rate of GTP–tubulins during assembly, which in turn depends on the lateral contacts of the GTP–tubulin heterodimer at the growing end^42^. Our results suggest that this effect is not significant in our A-lattice seam-enriched MTs. However, the fraction of A-lattice in the dynamic MTs extending from the A-lattice-enriched seeds is unknown and at least some MTs and seeds will be below the population mean of 50% A-lattice^29^, in which case any effect of A-lattice inclusions on growth rate might be hard to detect. GMPCPP MTs do not undergo dynamic instability^3^ and GMPCPP tubulin can act as a stabilizing cap on GDP-MT ends^12^, suggesting that GMPCPP MTs are a model for the stabilizing MT cap. Our observations therefore suggest that increasing the A-lattice content destabilizes the GTP caps in MTs. We propose that the stability of lateral PF contacts in the GTP cap of dynamic MTs would be similarly reduced, and that this increases the catastrophe frequency. We find that increased A-lattice content correlates to decreased MT stability, but we cannot yet directly determine how the number and distribution of A-lattice seams or the distribution of lattice defects contribute to the overall stability of the GTP cap and GDP core of the MT.

Increased A-lattice content causes a dramatic decrease in GMPCPP MT lattice stability yet our observations suggest only a modest effect on catastrophe frequencies in dynamic MTs. Several factors may have led us to underestimate the influence of A-lattice seams on dynamics. Our structural study^29^ showed that the seeds contain a distribution of A-lattice content from normal B-lattice with a single A-lattice seam through to 100% A-lattice, with most MTs containing a mixture averaging 50% A-lattice. Since the A-lattice seeds are unstable in solution after removal of the Mal3 our preparation procedure may bias the seeds towards more stable forms with lower A-lattice content. When we examined the catastrophe frequency distribution for the A-lattice-enhanced seeds in the population we found that many had catastrophe frequencies indistinguishable from the B-lattice (single A-lattice seam) seeds (Fig. 2i). However, seeds were also present in the population with increased catastrophe frequency, which correlated with increased minus-end shrinkage rates (Fig. 2j). This suggests that our A-lattice-enriched seeds are a mixed population, with many indistinguishable from normal B-lattice MTs. In these experiments the control MT seeds are B-lattice with a single A-lattice seam rather than pure B-lattice MTs, which may also reduce the apparent effect of additional MT seams.

It is also unclear how efficiently the A-lattice content of the nucleating seeds is propagated into the dynamic MTs as they extend from the seeds. We observed rapid short fluctuating MT growths from seeds that we termed MT dithering. MT dithering increased dramatically on MT seeds with increased A-lattice content. We speculate that MT dithering arises when the growing MT contains a high fraction of A-lattice, such that catastrophe occurs very shortly after growth has initiated. Consistent with this idea, time spent dithering increases with increasing Mal3FL concentrations during seed assembly (Fig. 3c, Table 1). If this is correct, it implies that those MTs that do succeed in growing under our conditions are selected for improved stability corresponding to a moderate A-lattice content. This in turn will lead us to underestimate the destabilizing effect of increased A-lattice content. Despite all these effects acting to reduce the apparent effect of the A-lattice enrichment on dynamics, it is nonetheless very clear that A-lattice enrichment enhances the catastrophe frequency at both ends of dynamic MTs.

Lastly, we observed that for dynamic MTs that were only attached to the surface via their seeds, A-lattice enrichment accelerated post-catastrophe shrinkage of the GDP core of the MT at the minus ends but not at the plus ends. We examined this further using our kinesin-clamp assay. We found that B-lattice GDP MTs with a single-seam were stabilized by binding to a rigor kinesin surface. In contrast, for MTs with extra A-lattice seams, the stabilizing effect of the rigor kinesin clamps was reduced so that the GDP lattice shrank even before the addition of ATP, with the plus end shrinking faster than the minus end. Thus, while a difference in the plus- and minus-end shrinkage rates is detectable when shrinkage is slowed by the kinesin clamps, in dynamic MTs not directly attached to the surface, the plus-end shrinkage rate is saturated and insensitive to changes in the lattice arrangement. Our overall conclusion is that like the GTP cap, the GDP core is also destabilized by A-lattice enrichment.

The influence of EB family proteins on MT dynamics remains highly controversial^43^. Our observations have some implications and we offer brief comments. Studies *in vivo* and *in vitro* have produced apparently contradictory observations (reviewed in ref. 44). *In vitro* studies show no apparent correlation of dynamics effects with either the proteins or experimental conditions employed (Supplementary Table 1). Furthermore, the observed *in vitro* effects often do not correspond to the *in vivo* effects of the same proteins. It may be that the main function of EB proteins *in vivo* is to tip-track and position other proteins that more directly affect MT dynamics^45^. The specific role of Mal3, the *S. pombe* EB, is at least equally controversial. *In vivo*, Mal3 inhibits MT catastrophe^46^. Our observations *in vitro* show that Mal3 in fact does not inhibit catastrophe, but rather masks it, by slowing subsequent MT shrinkage and promoting rescue^37^. This is a commonly though not universally reported feature of EB activity *in vitro* (Supplementary Table 1). Some studies have however found completely the opposite, that the effect of Mal3 *in vitro* is to increase catastrophe frequency^47^. We have here carried out extensive control experiments that confirmed our earlier observations that our Mal3 construct does not increase catastrophe frequency (Table 2). This indicates that our observed effects do not result from residual Mal3 in our assays. Our Mal3 constructs retain a his tag, which is absent in the construct used by Bieling *et al.*^47^. The his tag can increase the affinity of EBs for MTs^48^ and this might account for some of the observed differences in behaviour, but there is no correlation in other EBs between the presence of the his tag and effects on MT dynamics *in vitro* (Supplementary Table 1). The binding of Mal3 to MTs is also controversial. Earlier EM structural studies show that Mal3-N143 binds pure A-lattice *S. pombe* and brain tubulin MTs assembled with GMPCPP or GDP^29^, while full-length Mal3 binds the A-lattice seam of B-lattice brain tubulin MTs assembled with GMPCPP or GDP^36^. In the study of Sandblad *et al.*^36^ B-lattice MTs with multiple A-lattice seams were also observed. However, a more recent EM study^38^ found that Mal3–N143 bound to brain tubulin GTPγS and GDP MTs only on the B-lattice and not at the A-lattice seam where the B-lattice-binding site they identified does not exist. In this case the discrepancy in whether Mal3 can bind A- or B-lattice cannot be explained by the His tag since the full-length Mal3 protein used by Sandblad *et al.*^36^ and the N143 truncation used by Maurer *et al.*^38^ both had the His tag removed. However, the His tag may explain the binding of Mal3–N143 to GMPCPP MTs observed by des Georges *et al.*^29^, which is weaker than binding to GTPγS MTs when the His tag is absent^49^. We can only speculate that some subtlety in experimental protocol gives rise to such radically different structural results. Possibly the higher affinity of His-tagged Mal3–N143 (ref. 29)^29^ and full-length dimeric Mal3 (ref. 36)^36^ enhance binding to the A-lattice. Alternatively, although all three studies co-assembled Mal3 and tubulin^29^,^36^,^38^ only in Maurer *et al.*^38^ were the co-assembled MTs nucleated by a pre-assembled GMPCPP stabilized seed. Our previous biochemical studies indicate that Mal3 can fully decorate both B- and A-lattice-enriched MTs^29^, although it is not clear whether Mal3 was binding to the same site in both cases. Mal3 binds more tightly to MT tips than to the MT lattice, indicating that it has at least two different modes of MT binding. The experiments of Maurer *et al.*^38^,^49^, using GTPgS MTs, may have isolated the tight tip-binding mode.

**Figure 7:**
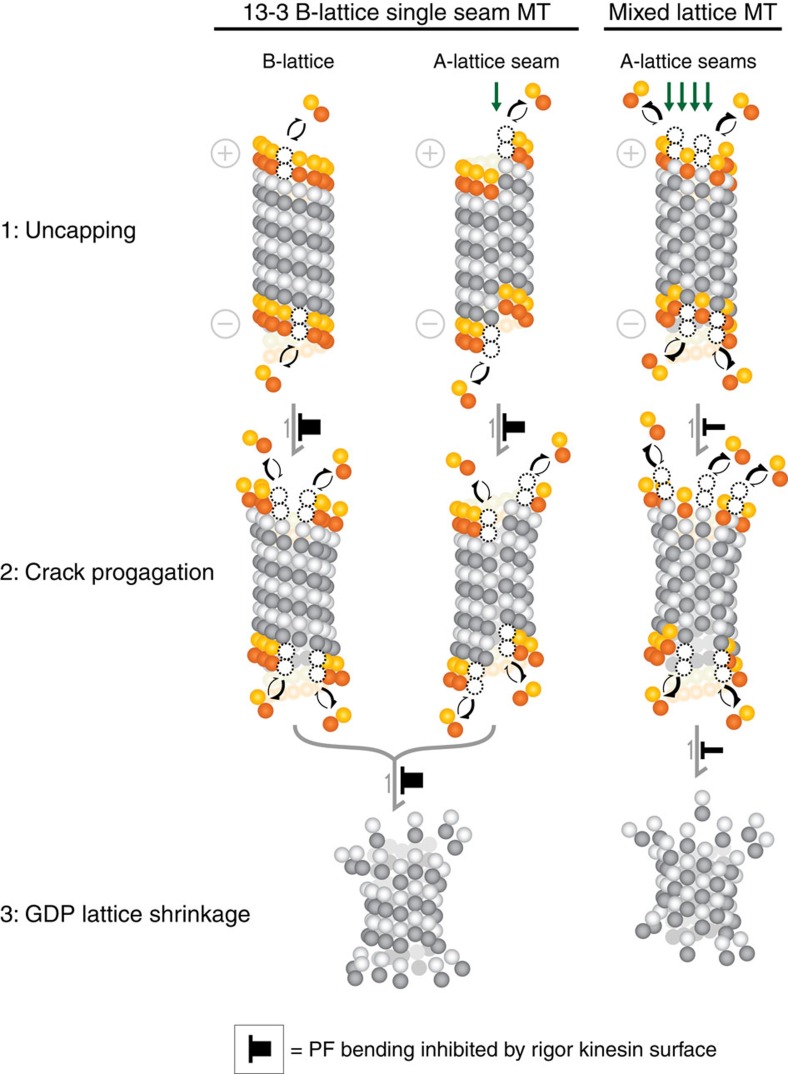
The role of A-lattice seams in MT catastrophe. In 13–3 B-lattice single-seam MTs PFs form strong lateral bonds (left panel) except at the A-lattice contacts of the MT seam (middle panel). B-lattice MTs enriched with A-lattice seams have more of the weaker contacts (right panel). (1) Uncapping: loss of GTP tubulin uncaps the MT. Detachment of tubulin from most of the B-lattice end is limited by the two strong lateral interactions on both sides of the heterodimer (left panel). Because of the spiral arrangement of heterodimers one heterodimer at the end will only make one lateral B-lattice contact (middle panel). At A-lattice seams this contact will be weaker and the heterodimer will detach more easily. In a MT enriched for A-lattice the MT end will become crenellated and more heterodimers will have only two weaker half lateral interactions, increasing their rate of detachment (right panel). (2) Crack propagation: crack formation breaks lateral contacts in both A- and B-lattices enhancing the dissociation of the GTP tubulin heterodimers in the cap as more heterodimers become exposed with fewer lateral contacts. Cracks propagate preferentially along the less stable lateral contacts between PFs in A-lattice seams. (3) GDP lattice shrinkage: shrinkage of GDP lattice proceeds via outward bending of the PFs in both the B-lattice (left hand) and A-lattice seams (middle, right hand panels). Weaker lateral contacts in A-lattice seams promote this process. At minus ends this process appears to limit shrinkage rates, possibly because of exposed GTP alpha tubulin. Plus ends expose GDP beta tubulin and PF unpeeling is then not limiting the depolymerization rate. Mechanical constraint of the MT, for example by rigor-bound kinesin motors attached to a surface, would inhibit both crack formation (step 2) and PF bending (step 3), inhibiting the transition to rapid post-catastrophe shrinkage of GDP-MT lattice (black top hats). Thus, catastrophe has two distinct steps: dissociation (or hydrolysis) of the GTP subunits forming the cap, followed by an allosteric change with bending of the PFs. A-lattice seams enhance both processes, so that seams promote both the loss of the GTP cap and the subsequent transition to rapid shrinkage.

To account for our observations, we envisage a multi-step model for catastrophe (Fig. 7), with two classes of processes that are sensitive to increased A-lattice content. First, we suppose that the dissociation rate of subunits from the MT tips is sensitive to the A-lattice seam content. In a 13–3 B-lattice MT with a single A-lattice seam (Fig. 7, left, centre) most terminal tubulin heterodimers are flanked by two heterodimers forming strong lateral contacts (Fig. 7, left). There are then two effects that tend to destabilize terminal heterodimer subunits. First, subunits at corners have only one lateral contact (centre), and will therefore be less stable than those with two lateral contacts. As additional A-lattice is introduced, the MT tip becomes crenellated (Fig. 7, right) and a second destabilizing effect arises. Increasing numbers of heterodimer subunits project from the tip by one monomer length and make β–α rather than β–β and α–α contacts. This will again increase their rate of detachment. Breaches in the stabilizing cap of GTP–tubulin are correspondingly more likely to occur. Potentially, the stabilizing GTP–tubulin cap is as small as a few layers of heterodimers. It has been proposed that only three breaches of the cap would be required to trigger catastrophe^16^. Our model envisages that seams produce a localized increase in GTP tubulin detachment rates and that this effect substantially enhances the probability of catastrophe while having only a very modest effect on the overall growth rate. This is consistent with our finding that overall growth rate of the MT is not significantly altered by the inclusion of extra A-lattice seams.

Second, we envisage that cracking of the lattice is sensitive to the A-lattice content of the lattice. Preferential cracking of the cap at the seams would reduce the number of lateral contacts on the subunits adjacent to the crack and increase curling of the PFs, further increasing their dissociation rate. Some recent modelling has proposed a role for cracks extending into the GDP core in promoting catastrophe^50^. Loss of the GTP cap via this cracking mechanism would be predicted to be blocked by clamping the MT to a surface using antibodies or rigor kinesin, thereby inhibiting PF unpeeling/curling^5^,^6^,^32^. Following loss of the stabilizing cap MTs normally transition to rapid shrinkage of the GDP–tubulin core. B-lattice single-seam dynamic MTs shrink slowly when stabilized by the rigor kinesin surface, indicating that shrinkage of the GDP core following catastrophe is inhibited by the kinesin surface clamp. Addition of ATP triggers immediate, rapid shrinkage. By contrast, A-lattice-enriched MTs are less well stabilized by the rigor kinesin surface. This suggests that additional A-lattice seams produce a delocalized destabilization of the entire tube, both in the cap and in the core. Thus, we propose that lateral inter-PF contacts are important in determining the stability of the MT lattice and its stabilizing cap, with the weaker A-lattice contacts of the seam having a particularly significant role.

Our data and our model suggest that the predominant 13–3 B-lattice MT geometry, incorporating a single A-lattice seam, may represent evolutionary tuning to provide a metastable structure, with the A-lattice seam as a structural mechanism to decrease the intrinsic stability of B-lattice MTs. The seam would also provide a potential hotspot for catastrophe, making MT dynamics more responsive to regulation. Regulation could involve proteins targeting the seam. Only Mal3 has so far been proposed as a putative seam-binding stabilizer^36^ and this role remains uncertain^38^. It will be interesting to investigate whether naturally occurring MTs lacking A-lattice seams^25^,^26^,^27^,^28^ are selected for specialized roles requiring less dynamic MTs.

## Methods

### Biochemical reagents

GTP, ATP and GMPCPP were purchased from Jena Biosciences (Germany), PIPES from Melford (UK) and other reagents from Sigma (UK) except where stated.

### Proteins

Pig brain tubulin^51^ was prepared by homogenization of six pig brains in 400 ml of homogenization buffer (100 mM K-PIPES (pH 6.8), 0.5 mM MgCl_2_, 2 mM EGTA, 0.1 mM EDTA, 1 mM Mg.ATP, 0.1 mM GTP, 1 mM DTT, 4 μM DCI, 10 μg ml^−1^ Leupeptin, 10 μg ml^−1^ Pepstatin, 1 μg ml^−1^ Aprotinin) and centrifuged at 35,800 *g* in an SLA1000 (Thermo) rotor for 50 min at 4 °C. The supernatant was adjusted to 33% (*v*/*v*) glycerol, 5 mM MgCl_2_, 1 mM GTP and incubated at 37 °C for 50 min, then centrifuged in an SLA1000 rotor at 35,800 *g* for 2.5 h at 35 °C. The pellet was resuspended in homogenization buffer at 4 °C, pelleted in a T865 (Thermo) rotor at 163,000 *g* for 1 h at 4 °C. The supernatant was adjusted to 33% (*v*/*v*) glycerol, 5 mM MgCl_2_, 1 mM GTP and incubated at 37 °C for 40 min, then centrifuged in a T865 (Thermo) rotor at 163 000 *g* for 1 h at 35 °C. The pellet was resuspended in 50 mM K-PIPES (pH 6.9), 1 mM EGTA, 0.2 mM MgCl_2_, 0.1 mM GTP, clarified by centrifugation in a TLA 100.3 rotor (Beckman) at 135,000 *g* for 30 min at 4 °C and loaded on a P11 phosphocellulose column (Whatman). Tubulin was eluted using 50 mM K-PIPES (pH 6.9), 1 mM EGTA, 0.2 mM MgCl_2_ and 10 μM GTP, then desalted into 100 mM K-PIPES (pH 6.9), 1 mM MgSO_4_, 2 mM EGTA and 20 μM GDP using a HIPREP 26/10 desalting column (GE healthcare). The tubulin was then aliquoted, flash-frozen and stored in liquid nitrogen. Pig tubulin concentration was determined using *E*_280_=105,838 M^−1^ cm^−1^.

Pig brain tubulins labelled with Alexa-488 and Alexa-680 fluorophores (Invitrogen) were prepared^52^ by resuspending MTs at 100 mg ml^−1^ in 0.1 M HEPES (pH 8.6), 1 mM MgCl_2_, 1 mM EGTA, 1 mM GTP, 40% (*v*/*v*) glycerol at 37 °C. Alexa Carboxylic acid succinimidyl ester dyes (40 mM) of Alexa-488 or Alexa-680 in DMSO were added to 4 mM final concentration, vortexed and incubated for 10 min at 37 °C, with vortexing every 2 min. Two volumes of 0.16 M MES (pH 6.8), 1 mM MgCl_2_, 1 mM EGTA, 1 mM GTP, 0.1 M K-glutamate, 40% (*v*/*v*) glycerol at 37 °C were added and vortexed. The MTs were pelleted through a 0.4 ml cushion of 80 mM MES (pH 6.8), 1 mM MgCl_2_, 1 mM EGTA, 1 mM GTP, 60% (*v*/*v*) glycerol in a TLA 120.2 rotor (Beckman) at 174,000*g* for 30 min at 35 °C. The pellet was washed with 80 mM MES (pH 6.8), 1 mM MgCl_2_, 1 mM EGTA and 1 mM GTP, at 37 °C then resuspended in the same buffer at 4 °C for 10 min and centrifuged at 170,000 *g* in a TLA120.2 rotor (Beckman) for 15 min at 4 °C. Glycerol (0.5 volume) was added to the supernatant, which was then incubated at 37 °C for 30 min. The MTs were again pelleted through the glycerol cushion, washed and resuspended in 80 mM MES (pH 6.8), 1 mM MgCl_2_, 1 mM EGTA and 1 mM GTP, at 4 °C, clarified by centrifugation at 170,000 *g* in a TLA120.2 rotor (Beckman) for 15 min at 4 °C, aliquoted, flash-frozen and stored in liquid nitrogen. Labelled tubulin concentrations (usually ∼200 μM) were calculated using a corrected OD_280_=OD_280measured_−(OD_495_ × 0.11) for Alexa-488 and corrected OD_280_=OD_280measured_−(OD_679_ × 0.05) for Alexa-680-labelled tubulin. The OD_280corrected_ was used to determine the pig tubulin concentration using *E*_280_=105,838 M^−1^ cm^−1^.

*S. pombe* single-isoform tubulin^29^,^53^ was prepared^54^ by grinding 500–600 g of *S. pombe* cell pellets in 1.8 l of 100 mM K-PIPES (pH 6.9), 10 mM MgSO_4_, 2 mM EGTA, 50 mM NaCl, 1 mM GTP, 5 mM DTT, 1 mM TAME, 1 μg ml^−1^ aprotinin, 1 μg ml^−1^ AEBSF, 1 μg ml^−1^ E-64, 1 μg ml^−1^ Pepstatin A, 1 μg ml^−1^ Chymostatin, 1 μg ml^−1^ Antipain, 2 μg ml^−1^ Leupeptin in a Dyno-Mill (Willy A. Bachofen) using 500 μm glass beads at 4 °C. After centrifugation at 24,400*g* in an SLA-3000 rotor (Thermo) for 30 min at 4 °C the supernatent was batch bound with gentle stirring to 180 g of equilibrated DE-52 resin (Whatman) at 4 °C for 30 min. After settling for 30 min the supernatant was discarded and the resin washed with 450 ml of DE-52 low-salt buffer (100 mM K-PIPES (pH 6.9), 10 mM MgSO_4_, 2 mM EGTA, 50 mM NaCl, 10% (*v*/*v*) glycerol, 0.1 mM GTP, 5 mM DTT, 1 μg ml^−1^ aprotinin, 1 μg ml^−1^ AEBSF, 1 μg ml^−1^ E-64, 1 μg ml^−1^ Pepstatin A, 1 μg ml^−1^ Chymostatin, 1 μg ml^−1^ Antipain, 2 μg ml^−1^ Leupeptin) and allowed to settle for 50 min. The supernatant was removed and the resin packed in an X50/30 column (GE Healthcare). Using an Akta purifier (GE Healthcare) the resin was washed with 3–4 column volumes of DE-52 low-salt buffer. The tubulin was eluted using a 0–60% gradient of high salt DE-52 buffer (100 mM K-PIPES (pH 6.9), 10 mM MgSO_4_, 2 mM EGTA, 1 M NaCl, 10% (*v*/*v*) glycerol, 0.1 mM GTP, 5 mM DTT, 1 μg ml^−1^ aprotinin, 1 μg ml^−1^ AEBSF, 1 μg ml^−1^ E-64, 1 μg ml^−1^ Pepstatin A, 1 μg ml^−1^ Chymostatin, 1 μg ml^−1^ Antipain, 2 μg ml^−1^ Leupeptin). Tubulin containing fractions were precipitated by adding 375 mg of ammonium sulphate per ml and incubating overnight at 4 °C. The precipitate was pelleted at 10,000 *g* in an SS34 rotor (Thermo) for 30 min at 4 °C then resuspended in 12 ml of resuspension buffer (100 mM K-PIPES (pH 6.9), 1 mM MgSO_4_, 2 mM EGTA, 0.1 mM GTP, 1 μg ml^−1^ aprotinin, 1 μg ml^−1^ AEBSF, 1 μg ml^−1^ E-64, 1 μg ml^−1^ Pepstatin A, 1 μg ml^−1^ Chymostatin, 1 μg ml^−1^ Antipain, 2 μg ml^−1^ Leupeptin) at 4 °C for 45 min. After centrifugation at 18,000 *g* in a microcentrifuge for 2 min at 4 °C the supernatant was desalted into resuspension buffer using a HiPrep 26/10 desalting column (GE Healthcare). Tubulin fractions were loaded onto a 5 ml HiTrap Q HP column (GE Healthcare) and washed with Q low-salt buffer (100 mM K-PIPES (pH 6.9), 1 mM MgSO_4_, 2 mM EGTA, 50 mM KCl, 50 μM GDP) then eluted using a 0–70% gradient of Q high-salt buffer (100 mM K-PIPES (pH 6.9), 1 mM MgSO_4_, 2 mM EGTA, 1 M KCl, 50 μM GDP). Tubulin-containing fractions were polymerized by adding GMPCPP to 0.6 mM, 1/10 vol of DMSO and 1/10 vol of glycerol then incubating for 1 h at 32 °C. MTs were pelleted at 124,000 *g* in a TLA100.3 rotor (Beckman) for 10 min at 32 °C and the pellet washed with 100 mM K-PIPES (pH 6.9), 1 mM MgSO_4_, 2 mM EGTA, 20 μM GMPCPP at 32 °C. Pellets were resuspended in 5 ml of 20 mM Na-PIPES (pH 6.9), 1 mM MgSO_4_, 5 mM CaCl_2_, 1 mM GDP at 32 °C for 10 min then incubated at 4 °C for 1 h with mixing. After centrifugation at 124,000 *g* in a TLA 100.3 rotor (Beckman) for 10 min at 4 °C the supernatant was loaded on a Superdex 200 XK16/60 gel filtration column (GE Healthcare) equilibrated with 100 mM K-PIPES (pH 6.9), 1 mM MgSO_4_, 2 mM EGTA, 150 mM NaCl, 50 μM GTP and eluted in the same buffer. The tubulin was then desalted into 100 mM K-PIPES (pH 6.9), 1 mM MgSO_4_, 2 mM EGTA, 20 μM GDP using a HiPrep 26/10 desalting column (GE Healthcare). The tubulin was then concentrated to 30–40 μM using a Vivaspin 15R 10,000 mwco Spin Concentrator (Viva products) at 2,675 *g* in a swing out rotor (Centra MP4R centrifuge) at 4 °C. The tubulin was aliquoted, flash-frozen and stored in liquid nitrogen. Tubulin concentration was measured using *E*_280_=108,390 M^−1^ cm^−1^.

Mal3-308 full-length and Mal3-143 encoding the N-terminal 143 amino acids of Mal3 were expressed, purified and quantified^29^. *E. coli* containing the pET vector expression constructs were grown to OD_600_=0.6 in LB, 100 μg ml^−1^ ampicillin at 37 °C, 250 r.p.m. Temperature was reduced to 20 °C, expression induced by adding IPTG to 0.5 mM and incubation continued at 20 °C, 250 r.p.m. for 6 h. Cells were pelleted at 1,500 × *g* in an SLA-3000 rotor (Thermo) at 4 °C for 15 min, then 12 g of cell pellet was resuspended in 60 ml of 15 mM Bicine (pH 8.0), 2 mM Mg Acetate, 0.4 M NaCl, 1 mM PMSF containing protease inhibitors (cOmplete EDTA-free, Roche). Cells were lysed by sonication (4 × 15 s at 4 °C) and the homogenate centrifuged in a T865 rotor (Thermo) at 206,000 *g* for 20 min at 4 °C. The supernatant was batch bound to 4 ml of Nickel-NTA resin (Qiagen) then packed in an XK 16/20 column (GE healthcare) and washed in 15 mM Bicine (pH 8.0), 2 mM MgAcetate, 0.4 M NaCl, 25 mM imadazole using an Akta purifier (GE Healthcare). Mal3 was eluted using 15 mM Bicine pH 8.0, 2 mM MgAcetate, 0.4 M NaCl, 100 mM imadazole then loaded on a Superdex 200 16/60 gel filtration column (GE Healthcare) equilibrated with 100 mM K-PEM (pH 6.9), 1 mM MgSO_4_, 2 mM EGTA, 100 mM NaCl and eluted in the same buffer. Aliquots were flash-frozen and stored in liquid nitrogen. Mal3-308 concentration was determined using *E*_280_=37,025 M^−1^ cm^−1^ and Mal3-143 using E_280_=32,555 M^−1^ cm^−1^.

### Flow cells

Coverslips were cleaned, silanized and flow cells assembled^37^. Coverslips (no 1.5, Menzel) were sonicated (600 W bath, Ultrawave) in a 1:1 (*v*/*v*) mixture of methanol and HCl for 30 min, rinsed by sonication in ultrapure water (18.2 Mohm, Elga) for 4 × 5 min, sonicated in 0.2 M KOH for 60 min, then washed by sonication in ultrapure water 5 × 5 min. Coverslips were spun (spin clean, Technical video) and then dried in an oven at 45 °C before silanization in 0.05% (*v*/*v*) dichlorodimethylsilane (DDS) in trichloroethylene (TCE) for 1 h. Coverslips were rinsed in methanol, then sonicated 6 × 5 min in methanol before being spun dry and stored in a desicator (Secador). Slides (Matsunami) were used without further processing. Flow cells were assembled by attaching the silanized coverslip to the slide using double-sided sticky tape (Scotch tape, 3M).

### GMPCPP MT seeds

Dual-labelled B-lattice single-seam MT seeds were assembled using 0.68 μM pig brain tubulin, 0.32 μM Alexa-680-labelled pig brain tubulin, 0.1 μM Alexa-488-labelled pig brain tubulin, 0.5 mM GMPCPP in K-PEM buffer (100 mM PIPES, 1 mM MgSO_4_, 2 mM EGTA (Fisher) adjusted to pH 6.9 with KOH^7^). Single-labelled B-lattice single-seam MT seeds were assembled using 1.0 μM pig brain tubulin, 0.1 μM Alexa-488-labelled pig brain tubulin, 0.5 mM GMPCPP in K-PEM buffer. For kinesin-clamp assays A-lattice-enriched seeds were formed by co-assembly of 1 μM pig brain tubulin, 0.1 μM Alexa-488-labelled pig brain tubulin with either 50 μM Mal3FL or 10 μM Mal3 N143 (ref. 29)^29^ in 0.5 mM GMPCPP, K-PEM. For MT dynamics assays A-lattice-enriched seeds were co-assembled from 1 μM pig brain tubulin, 0.1 μM Alexa-488-labelled pig brain tubulin with either 5 or 50 μM Mal3FL^29^ in 0.5 mM GMPCPP, K-PEM. The reaction mixes were incubated at 4 °C for 10 min to allow the GMPCPP to bind to the tubulin. Seeds were then polymerized by incubation at 37 °C for 60 min. Seeds were pelleted through a cushion of 60% (*v*/*v*) Glycerol in K-PEM at 90,000 *g* in a TLA100 rotor at 35 °C for 5 min using a Beckman Optima ultracentrifuge. The supernatant was discarded and the seeds resuspended in K-PEM buffer at 25 °C.

### Kinesin clamp MT gliding assay

A flow cell was directly coated with rat Kinesin-1 rK430GST^55^ (a gift from Isabel Crevel). Unbound kinesin was flushed out using K-PEM. GMPCPP MT seeds were flowed in and bound to the Kinesin for 10 min. Unbound MTs and, in A-lattice experiments, Mal3 was removed by flushing with K-PEM. Motility was initiated by flowing in 1 mM Mg.ATP, GCO oxygen scavenger (8 μg ml^−1^ catalase, 4.5 mg ml^−1^ glucose, 38 U ml^−1^ glucose oxidase), 1% (*v*/*v*) 2-mercaptoethanol in K-PEM.

### GDP-MT kinesin-clamp assay

B-lattice single-seam GDP *S. pombe* MTs were assembled by incubating 10 μM *S. pombe* tubulin, 1 mM GTP in K-PEM at 37 °C for 30 min. A-lattice MTs were assembled by incubating 5 μM *S. pombe* tubulin, 5 μM Mal3FL, 1 mM GTP in K-PEM at 37 °C for 30 min. MTs were then flushed into a flow cell coated with rKin430GST. After attachment for 10 min the flow cell was flushed with K-PEM buffer to remove unattached MTs and Mal3. B-lattice motility was examined by flushing in 1 μM Mg.ATP, GCO oxygen scavenger (8 μg ml^−1^ catalase, 4.5 mg ml^−1^ glucose, 38 U ml^−1^ glucose oxidase) in K-PEM.

### Segmented GMPCPP MT seeds

A-lattice GMPCPP MT seeds with alternating segments of fluorescent labelling (Lemur tail patterned)^52^ were assembled. First Alexa-680-labelled seeds were formed by incubating 1 μM pig brain tubulin containing 30% Alexa-680 and 10% Alexa-488-labelled subunits in 50 μM Mal3FL, 0.5 mM GMPCPP, K-PEM (100 mM K-PIPES (pH 6.9), 1 mM MgSO_4_, 2 mM EGTA) at 4 °C for 20 min then 37 °C for 60 min. An extension mix of 1 μM pig brain tubulin. containing 10% Alexa-488-labelled subunits in 50 μM Mal3FL, 0.5 mM GMPCPP, K-PEM was incubated at 4 °C for 20 min then pre-warmed at 30 °C for 10 min. Alexa-680 seeds (1/10 volume) were added to the extension mix and incubated at 30 °C for 15 min and then 37 °C for 45 min. The MTs were pelleted at 90,000 *g* in a TLA100 rotor (Beckman) for 5 min at 35 °C then resuspended in K-PEM. The MTs were incubated at room temperature before use to allow them to anneal to create the ‘lemur tail’ pattern.

### MT dynamics assay

A flow cell was coated with Anti-Alexa-488 antibodies (Molecular Probes) diluted 10-fold with K-PEM, then GMPCPP Alexa-488-labelled B-lattice single-seam or A-lattice-enriched pig tubulin MT seeds were added. Flow cells were flushed with K-PEM buffer to remove unbound seeds and, for A-lattice-enriched seeds, any residual Mal3 used for co-assembly. Dynamic MTs were assembled from the seeds using unlabelled 5.5 μM *S. pombe* single-isoform tubulin, 1 mM GTP and GCO oxygen scavenger in K-PEM.

### Microscopy and analysis

A Nikon E800 fluorescence microscope with a customized dark-field illumination system was used for image capture^37^. This was mounted in a custom box with a heater to maintain the temperature at 25 °C (Air-Therm ATX, WPI). A Nikon Plan Fluor × 100 0.5–3 NA variable iris objective lens and × 1.25 intermediate magnification were used and images captured by an EM-CCD (iXon^EM^+DU-897E, Andor). Epifluorescence illumination used a stabilized mercury lamp (X-cite exacte, Lumen Dynamics) coupled to the microscope via a light pipe. For dark-field illumination a 100-W mercury lamp was connected via a fibre optic light scrambler (Technical video), cold mirror and GIF 500–568 nm band-pass filter (Nikon). Fluorescence excitation and emission filters (Chroma) E460SPUVv2 and D525/20m for Alexa-488 together with S654/24x and S710/60m for Alexa-680 plus ND filters were mounted in motorized filter wheels (LEP MAC5000). An FF502/670-Di01 dichroic mirror (Semrock) permitted fluorescence and dark-field imaging. Electronic shutters were used to rapidly switch between fluorescence and epifluorescence modes. Metamorph software (Molecular Devices) was used to control the microscope and camera. Images for GTP dynamics were captured at 0.5 s intervals using 100 ms exposures at a spatial resolution of 0.128 μm pixel^−1^. GMPCPP kinesin-clamp assays used 1 s intervals and 0.128 μm pixel^−1^.

Metamorph software was used to generate kymographs of dynamic MTs. A custom macro GetEdge was used in ImageJ (Rasband, W.S., ImageJ, U. S. National Institutes of Health, Bethesda, Maryland, USA, http://imagej.nih.gov/ij/, 1997–2012) to automatically digitize MT end locations from the Kymographs^37^. The MT end positions were plotted against time and manually analysed to identify regions of growth and shrinkage. Growth and shrinkage rates were determined using linear regression fits. MT catastrophe frequencies were calculated as the total number of catastrophe events divided by the total time that MTs were observed in growth. Rescue frequencies were the total number of rescue events divided by the total time MTs were shrinking. Regrowth from the MT seed was not counted as a rescue.

### Statistics

Statistical tests were carried out using Prism (Graphpad) or Minitab software.

## Author contributions

M.K. and D.R.D. carried out the experiments. M.K., D.R.D. and R.A.C. devised and analysed experiments and prepared the manuscript.

## Additional information

**How to cite this article:** Katsuki, M. *et al.* Ectopic A-lattice seams destabilize microtubules. *Nat. Commun.* 5:3094 doi: 10.1038/ncomms4094 (2014).

## Supplementary Material

Supplementary Table and References Supplementary Table 1 and Supplementary References

Supplementary Movie 1 B-lattice MTs (right hand panel B) assembled from pig brain tubulin labelled with Alexa-480 fluorescent dye and GMPCPP, and A-lattice enriched MTs (left hand panel A-rich) co-assembled from pig brain tubulin labelled with Alexa-488, Mal3-N143 (CH domain) and GMPCPP were added to flow cells containing rat kinesin-1 motor protein dimers. Flow cells were flushed with buffer to remove Mal3 and unbound MTs. Buffer containing ATP was added and MT motility observed by timelapse epi-fluorescence microscopy. A-lattice enriched MTs shrink faster than B-lattice MTs. Time is in minutes: seconds. Scale bar is 10 μm.

Supplementary Movie 2 B-lattice MTs (red) were assembled using pig brain tubulin labelled with Alexa-680 and Alexa-480 fluorescent dyes and GMPCPP, and A-lattice enriched MTs (green) co-assembled with pig brain tubulin labelled with Alexa-488, Mal3FL and GMPCPP. After separate assembly reactions the B and A-lattice enriched MTs were mixed and added to a flow cell coated with rat kinesin-1 motor protein dimers. The flow cell was flushed with buffer to remove Mal3 and unbound MTs. Buffer containing ATP was added and MT motility observed by timelapse epi-fluorescence microscopy. A-lattice enriched MTs shrink faster than B-lattice MTs. Time is in minutes: seconds:milliseconds. Scale bar is 15 μm.

Supplementary Movie 3 B-lattice MT seeds (lower panel red outline) were assembled from pig brain tubulin labelled with Alex-488 fluorescent dye and GMPCPP. A-lattice enriched MT seeds (upper panel green outline) were co-assembled from pig brain tubulin labelled with Alexa-488, Mal3FL and GMPCPP. Seeds were attached to flow cells coated with anti-Alexa488 antibodies. Flow cells were flushed with buffer to remove Mal3 and unbound MTs. Buffer containing unlabelled S. *pombe* single isoform tubulin and GTP was added and MT dynamics observed using timelapse epifluorescence (for seeds) and darkfield (for dynamic unlabelled S. *pombe* MTs) microscopy. The MTs grown from the A-lattice enriched seeds had a higher catastrophe frequency and the minus ends a faster shrinkage rate. Green, Fluorescent MT seed; Grey, unlabelled dynamic MT; Yellow triangle, faster growing MT plus end. Time is in minutes: seconds. Scale bar is 3 μm.

Supplementary Movie 4 A-lattice enriched MT seeds (green) were co-assembled from pig brain tubulin labelled with Alexa-488, Mal3FL and GMPCPP. Seeds were attached to flow cells coated with anti-Alexa488 antibodies. Flow cells were flushed with buffer to remove Mal3 and unbound MTs. Buffer containing GTP and unlabelled S. *pombe* single isoform tubulin (grey) was added and MT dynamics observed using timelapse epifluorescence (for seeds) and darkfield (for dynamic unlabelled S. *pombe* MTs) microscopy. MTs grown from the A-lattice enriched seeds had frequent episodes of transient short MT growth, which we have named piku-piku or MT dithering (blue label). Time is in minutes. Scale bar is 5 μm.

## Figures and Tables

**Table 1:** Influence of ectopic A-lattice seams on MT dynamics.

	**Growth (mean±s.e.m. (*****n*****) nm s**^**−1**^**)**	**Shrinkage (mean±s.e.m. (*****n*****) nm s**^**−1**^**)**	**Catastrophe (frequency±CL (*****n*****) min**^**−1**^**)**	**Growth %**	**Shrinkage %**	**Dithering %**
*Plus end*						
B-lattice	5.86±0.19 (85)	158.5±6.0 (77)	0.25±0.20/0.29 (135)	92.1	1.9	6.0
A-lattice (5 μM Mal3FL seed)	6.22±0.15 (158)	156.5±4.48 (124)	0.33±0.29/0.38 (243)	90.5	2.4	7.1
A-lattice (50 μM Mal3FL seed)	5.27±0.38 (27)	139.3±10.7 (15)	0.35±0.27/0.46 (54)	74.7	1.8	23.5
						
*Minus end*						
B-lattice	3.62±0.16 (25)	90.9±10.8 (7)	0.040±0.03/0.07 (25)	99.2	0.6	0.2
A-lattice (5 μM Mal3FL seed)	4.11±0.11 (57)	140.6±10.2 (23)	0.080±0.06/0.10 (54)	97.4	1.1	1.5
A-lattice (50 μM Mal3FL seed)	3.55±0.33 (10)	130±20.1 (6)	0.12±0.07/0.19 (19)	98.3	1.7	0.0

MT dynamics parameters were determined from plots of MT length against time automatically generated from kymographs of dynamic (GTP/GDP) MTs (Fig. 2). Mean rates of growth and shrinkage were compared by one-way ANOVA and Tukey post test. The means of plus end growth rates (F (2, 267)=3.40, *P*=0.035), minus-end growth rates (F (2, 89)=3.82, *P*=0.026) and minus-end shrinkage rates (F (2, 29)=3.605, *P*=0.04) were significantly different, whilst plus end MT shrinkage rates were not (F (2, 213)=0.93, *P*=0.40). Using Tukey post test for pair-wise comparisons the differences in mean growth were significant (at *P*<0.05) between the two A-lattice seeds (5 μM Mal3FL was 1.2 × faster growth than 50 μM), but not between the A-lattice and B-lattice single-seam seeds. Minus-end shrinkage with A-lattice 5 μM Mal3FL seeds was significantly faster than from the B-lattice seeds. The Tukey test found no pair-wise significant differences in the minus-end growth rates at *P*<0.05. Total observation times were 598.4, 811.4 and 203.7 min at the plus end and 566.7, 703.8 and 158.5 min at the minus ends for B-lattice single-seam and A-lattice-enriched assembled from 5 and 50 μM Mal3FL seeds respectively. CL, Poison confidence limits, *P*=0.05.

**Table 2:** Control experiments: effect of Mal3 on B-lattice single seam MT dynamics.

**Mal3 (nM)**	**Growth (mean±s.e.m. (*****n*****), nm s**^**−1**^**)**	**Shrinkage (mean±s.e.m. (*****n*****), nm s**^**−1**^**)**	**Catastrophe (frequency±CL (*****n*****), min**^**−1**^**)**	**Rescue (frequency±CL (*****n*****), min**^**−1**^**)**
*Plus end*				
0	7.44±0.44 (148)	227.6±14.0 (136)	0.43±0.36/0.51 (137)	0.29±0.06/0.85 (3)
1	7.25±0.27 (138)	240.4±14.3 (126)*	0.49±0.41/0.58 (129)	0.00±0.00/3.69 (0)
10	6.03±0.29 (88)	316.0±26.4 (84)	0.48±0.38/0.59 (85)	0.22±0.01/1.22 (1)
50	7.20±0.53 (97)	213.2±17.5 (88)	0.48±0.38/0.59 (82)	0.88±0.32/1.92 (6)
100	9.12±0.38 (116)*	107.6±7.5 (108)*	0.56±0.46/0.68 (107)	4.33±3.44/5.37 (82)†
				
*Minus end*				
0	2.90±0.27 (71)	109.3±8.3 (59)	0.18±0.14/0.23 (57)	2.26±1.68/2.98 (50)
1	3.17±0.33 (63)	113.4±9.1 (53)	0.22±0.17/0.29 (53)	2.75±1.63/4.34 (18)
10	2.10±0.59 (17)	115.4±24.3 (14)	0.15±0.08/0.26 (14)	3.84±1.24/8.95 (5)
50	2.94±0.78 (39)	66.2±10.3 (58)*	0.24±0.17/0.34 (33)	0.59±0.40/0.84 (30)
100	1.53±0.25 (14)	60.6±9.5 (5)	0.03±0.01/0.06 (5)	5.52±1.79/12.89 (5)

Dynamic *S. pombe* microtubules were grown from GMPCPP stabilized B-lattice single-seam brain tubulin microtubule seeds in the presence of low concentrations of full-length dimeric Mal 3 protein and the microtubule dynamics parameters were determined.

^*^Growth and shrinkage rates significantly different from 0 nM Mal3 control determined by one-way ANOVA, and Tukey post test (at P< 0.05). Plus end growth (F (4, 582)=6.92, *P*< 0.0001) and shrinkage rates (F (4, 537)=20.14, *P*< 0.0001) contain significant differences. Only minus end shrinkage rates are significantly different (F (4, 184)=4.46, *P*=0.0018) with no differences between growth rates (F (4, 199)=1.13, *P*=0.34).

^†^Rescue frequencies significantly different from 0 nM Mal3 control determined by the confidence limits assuming a Poisson distribution (*P*=0.05). No significant differences in catastrophe frequency were observed.

**Table 3:** Mean shrinkage rates of GDP MTs.

	**ATP (μM)**	**Fast (plus) end (nm s**^**−1**^**±s.e.m. (*****n*****))**	**Slow (minus) end (nm s**^**−1**^**±s.e.m. (*****n*****))**
A-lattice (Mal3FL)	0	10.68±0.30 (5)	2.78±0.42 (5)
B-lattice	0	2.68±0.73 (5)	0.34±0.13 (5)
B-lattice	1	55.14±5.12 (5)	7.06±2.82 (5)

Mean shrinkage rates of GDP MTs held in a rigor kinesin clamp were compared before and after the release of the clamp by addition of 1 μM ATP. The shrinkage at the plus end was 29 times faster in the A-lattice enriched microtubules and significantly different from the B-lattice (*t*-test, *P*<0.0001). The slow ends of A-lattice-enriched MTs also shrank significantly faster than in the B-lattice MTs by 8 times (*t*-test with Welch’s correction for unequal variances, *P*=0.0054).
