# Supplementary material for: Ectopic A-lattice seams destabilize microtubules
Source: Nat Commun. 2014 Jan 27;5:3094. doi: 10.1038/ncomms4094 (PMC3921467; doi:10.1038/ncomms4094)
Supplement: Supplementary Table and References — Supplementary Table 1 and Supplementary References [file ncomms4094-s1.pdf]

Supplementary Information

Study	EB	Tag	EB nM	Tubulin	Tubulin µM	$MgCl_2$ mM	Temp °C	Growth	Shrinkage	Cat	Res
Vitre et al[39]	EB1 (Mm)	His	500 - 2000	mammalian brain	10	1	36	2x	-2x	2x	8x
Manna et al[56]	EB1 (Hs)	cleaved	500 - 2000	mammalian brain	20	1.4	30	1.2x	-2x	-2x	1.5x
Dixit et al[57]	EB1 (Hs)	cleaved	250	mammalian brain	22	2	22	~	1.1x	~	~
Li et al[58]	EB1 (Dm)	cleaved	100 - 800	mammalian brain	15	4	25	1.3x	~	~*	~
Komarova et al[45]	EB3 (Hs)	cleaved	200 - 1000	mammalian brain	15	4	25	5x	1.5x	9x	3x
Bieling et al[47]	Mal3 (Sp)	cleaved	200	mammalian brain	10	6	30	~	~	3x	~**
Katsuki et al[37]	Mal3 (Sp)	His	50 -500	*S. pombe*	4.5	1	25	~	-3x	~	6x
This study	Mal3 (Sp)	His	2 - 200	*S. pombe*	4.5	1	25	1.2x	-2x	~	15x

Supplementary Table 1 EB protein effects on microtubule dynamics in vitro

A summary of published observations on the effect of EB family proteins on microtubule dynamics in vitro. All significant changes in rates relative to tubulin only controls are given ($p < 0.05$).
*No change in catastrophe frequency was found when all data was averaged. However Li et al[58] report that if the increase in catastrophe frequency of tubulin plus EB1 compared to tubulin alone is calculated in individual experiments and then averaged they found a 1.5x increase in catastrophe frequency.
**No rescue events were observed in any conditions.
~: no significant change compared to tubulin only ($p > 0.05$). Mm: *Mus musculus*, Hs: *Homo sapiens*, Sp: *Schizosaccharomyces pombe.*
increase, *decrease in rate (slower growth or shrinkage). The largest change relative to the control is given.*

Supplementary references

56. Manna, T., Honnappa, S., Steinmetz, M.O. & Wilson, L. Suppression of microtubule dynamic instability by the +TIP protein EB1 and its modulation by the CAP-Gly domain of p150glued. *Biochemistry* **47**, 779-86 (2008).

57. Dixit, R. et al. Microtubule plus-end tracking by CLIP-170 requires EB1. *Proc Natl Acad Sci U S A* **106**, 492-7 (2009).

58. Li, W. et al. Reconstitution of dynamic microtubules with Drosophila XMAP215, EB1, and Sentin. *J Cell Biol* **199**, 849-62 (2012).
